# *N*′-[(*Z*)-(1,5-Dimethyl-3-oxo-2-phenyl-2,3-dihydro-1*H*-pyrazol-4-yl)methyl­idene]-2-hy­droxy­benzohydrazide

**DOI:** 10.1107/S1600536812000402

**Published:** 2012-01-11

**Authors:** Muhammad Aslam, Itrat Anis, Nighat Afza, Ejaz, Islam Ullah Khan, Muhammad Nadeem Arshad

**Affiliations:** a Pakistan Council of Scientific and Industrial Research Laboratories Complex, Karachi 75280, Pakistan; b Department of Chemistry, University of Karachi, Karachi 75270, Pakistan; c Materials Chemistry Laboratory, Department of Chemistry, GC University, Lahore 54000, Pakistan; d Department of Chemistry, University of Gujrat, Gujrat 50781, Pakistan

## Abstract

In the title compound, C_19_H_18_N_4_O_3_, the pyrazole ring is oriented at dihedral angles of 41.12 (7) and 12.25 (10)°, respectively, with respect to the planes of the phenyl and benzene rings. Intra­molecular N—H⋯O and O—H⋯O hydrogen bonds generate seven- and six-membered *S*(7) and *S*(6) ring motifs, respectively.

## Related literature

For the biological activity of Schiff bases, see: Lau *et al.* (1999 ▶); More *et al.* (2002 ▶); Safwat *et al.* (1988 ▶); Sharma *et al.* (1998 ▶); Pandeya *et al.* (1999 ▶). For graph-set notation, see: Bernstein *et al.* (1995 ▶).
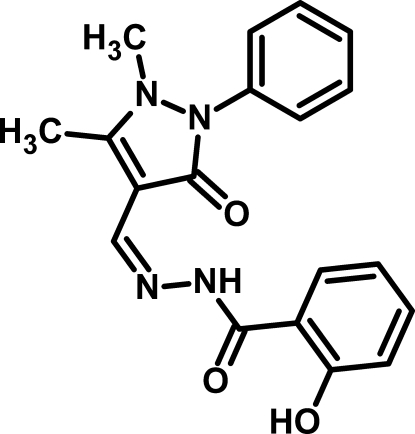


## Experimental

### 

#### Crystal data

C_19_H_18_N_4_O_3_
*M* *_r_* = 350.37
Monoclinic, 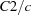

*a* = 25.2357 (6) Å
*b* = 8.5624 (2) Å
*c* = 16.0329 (4) Å
β = 104.048 (1)°
*V* = 3360.75 (14) Å^3^
*Z* = 8
Mo *K*α radiation
μ = 0.10 mm^−1^
*T* = 296 K
0.22 × 0.06 × 0.05 mm

#### Data collection

Bruker Kappa APEXII CCD diffractometer
Absorption correction: multi-scan (*SADABS*; Bruker, 2007 ▶) *T*_min_ = 0.980, *T*_max_ = 0.995
16063 measured reflections
4174 independent reflections
2653 reflections with *I* > 2σ(*I*)
*R*_int_ = 0.029

#### Refinement

*R*[*F* ^2^ > 2σ(*F* ^2^)] = 0.043
*wR*(*F* ^2^) = 0.119
*S* = 1.02
4174 reflections
243 parameters
H atoms treated by a mixture of independent and constrained refinement
Δρ_max_ = 0.16 e Å^−3^
Δρ_min_ = −0.19 e Å^−3^

### 

Data collection: *APEX2* (Bruker, 2007 ▶); cell refinement: *SAINT* (Bruker, 2007 ▶); data reduction: *SAINT*; program(s) used to solve structure: *SHELXS97* (Sheldrick, 2008 ▶); program(s) used to refine structure: *SHELXL97* (Sheldrick, 2008 ▶); molecular graphics: *ORTEP-3 for Windows* (Farrugia, 1997 ▶) and *PLATON* (Spek, 2009 ▶); software used to prepare material for publication: *WinGX* (Farrugia, 1999 ▶) and *PLATON*.

## Supplementary Material

Crystal structure: contains datablock(s) I, global. DOI: 10.1107/S1600536812000402/is5039sup1.cif

Structure factors: contains datablock(s) I. DOI: 10.1107/S1600536812000402/is5039Isup2.hkl

Supplementary material file. DOI: 10.1107/S1600536812000402/is5039Isup3.cml

Additional supplementary materials:  crystallographic information; 3D view; checkCIF report

## Figures and Tables

**Table 1:** Hydrogen-bond geometry (Å, °)

*D*—H⋯*A*	*D*—H	H⋯*A*	*D*⋯*A*	*D*—H⋯*A*
O3—H3*O*⋯O2	0.95 (2)	1.62 (2)	2.5076 (17)	155 (2)
N4—H4*N*⋯O1	0.978 (18)	1.763 (18)	2.7140 (16)	163.3 (16)

**Fig. 1.**
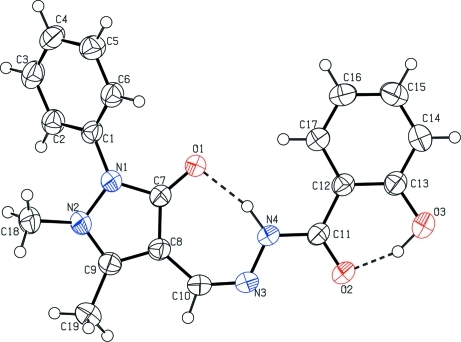
The molecular structure of the title compound, with 50% displacement ellipsoids. The hydrogen bonds are shown with dashed lines.

## supplementary crystallographic information

### Comment

The Schiff base ligands have importance for elucidating the mechanism of racemization and transamination reactions in biological systems (Lau *et al.*, 1999) and exhibit remarkable biological activities such as antibacterial (More *et al.*, 2002), antifungal (Safwat *et al.*, 1988), anticancer (Sharma *et al.*, 1998) and anti HIV activities (Pandeya *et al.*, 1999). Such applications lead us to report the synthesis and characterization of Schiff base ligand, *N*'-((1,5-dimethyl-3-oxo-2-phenyl-2,3-dihydro-1*H*-pyrazol-4-yl) methylene)-2-hydroxybenzohydrazide.

In the crystal structure of title compound, two nitrogen and three carbon atoms of pyrazole ring are *sp*^2^ hybridized, whose ring shows an r.m.s. deviation of about 0.1 Å with the maximum deviation of from N1 [0.1256 (12) Å] and C8 [0.1237 (10) Å]. The molecule contains N—H and O—H groups but no classical intermolecular hydrogen bonding has been observed. Only intramolecular hydrogen bonding produces six-membered *S*(6) and seven-membered *S*(7) (Bernstein *et al.*, 1995) ring motifs through O—H···O and N—H···O interactions (Table 1 and Fig. 1). The two ring motifs O1/C7/C8/C10/N3/N4/H4N and O2/C11/C12/C13/O3/H3O are inclined at a dihedral angle of 3.46 (4)°. The phenyl ring (C1–C6) is twisted at 41.12 (7) and 41.64 (7)°, respectively, with the pyrazole ring and *o*-hydroxy benzoyl ring (C12–C17).

### Experimental

1,5-Dimethyl-3-oxo-2-phenyl-2,3-dihydro-1*H*-pyrazole-4-carbaldehyde (1 mol) and 2-hydroxybenzohydrazide (1 mol) were added in 50 ml ethanol and add 3–4 drops of conc. H_2_SO_4_. The mixture was refluxed with stirring for 5 h at 70 °C on water bath. The reaction mixture was kept at room temperature overnight and white crystals were obtained. These were filtered, washed with cooled methanol, dried and recrystallized from methanol.

### Refinement

C-bound H atoms were positioned with idealized geometry with C—H = 0.93 Å for aromatic and C—H = 0.96 Å for methyl group, and were refined using a riding model with *U*_iso_(H) = 1.2*U*_eq_(C) for aromatic or 1.5*U*_eq_(C) for methyl carbon atoms. The N and O-bound H atoms were located in a difference Fourier map and the positional parameters were refined with *U*_iso_(H) = 1.2*U*_eq_(N) or 1.5*U*_eq_(O); the refined distances are N—H = 0.978 (18) Å and O—H = 0.95 (2) Å

### Crystal data

**Table:**

C_19_H_18_N_4_O_3_	*F*(000) = 1472
*M**_r_* = 350.37	*D*_x_ = 1.385 Mg m^−^^3^
Monoclinic, *C*2/*c*	Mo *K*α radiation, λ = 0.71073 Å
Hall symbol: -C 2yc	Cell parameters from 3642 reflections
*a* = 25.2357 (6) Å	θ = 2.5–25.1°
*b* = 8.5624 (2) Å	µ = 0.10 mm^−^^1^
*c* = 16.0329 (4) Å	*T* = 296 K
β = 104.048 (1)°	Needle, colorless
*V* = 3360.75 (14) Å^3^	0.22 × 0.06 × 0.05 mm
*Z* = 8	

### Data collection

**Table:**

Bruker Kappa APEXII CCD diffractometer	4174 independent reflections
Radiation source: fine-focus sealed tube	2653 reflections with *I* > 2σ(*I*)
graphite	*R*_int_ = 0.029
φ and ω scans	θ_max_ = 28.3°, θ_min_ = 2.8°
Absorption correction: multi-scan (*SADABS*; Bruker, 2007)	*h* = −33→32
*T*_min_ = 0.980, *T*_max_ = 0.995	*k* = −11→11
16063 measured reflections	*l* = −21→21

### Refinement

**Table:**

Refinement on *F*^2^	Primary atom site location: structure-invariant direct methods
Least-squares matrix: full	Secondary atom site location: difference Fourier map
*R*[*F*^2^ > 2σ(*F*^2^)] = 0.043	Hydrogen site location: inferred from neighbouring sites
*wR*(*F*^2^) = 0.119	H atoms treated by a mixture of independent and constrained refinement
*S* = 1.02	*w* = 1/[σ^2^(*F*_o_^2^) + (0.0527*P*)^2^ + 0.9211*P*] where *P* = (*F*_o_^2^ + 2*F*_c_^2^)/3
4174 reflections	(Δ/σ)_max_ < 0.001
243 parameters	Δρ_max_ = 0.16 e Å^−^^3^
0 restraints	Δρ_min_ = −0.19 e Å^−^^3^

### Special details

**Table:**

Geometry. All e.s.d.'s (except the e.s.d. in the dihedral angle between two l.s. planes) are estimated using the full covariance matrix. The cell e.s.d.'s are taken into account individually in the estimation of e.s.d.'s in distances, angles and torsion angles; correlations between e.s.d.'s in cell parameters are only used when they are defined by crystal symmetry. An approximate (isotropic) treatment of cell e.s.d.'s is used for estimating e.s.d.'s involving l.s. planes.
Refinement. Refinement of *F*^2^ against ALL reflections. The weighted *R*-factor *wR* and goodness of fit *S* are based on *F*^2^, conventional *R*-factors *R* are based on *F*, with *F* set to zero for negative *F*^2^. The threshold expression of *F*^2^ > σ(*F*^2^) is used only for calculating *R*-factors(gt) *etc*. and is not relevant to the choice of reflections for refinement. *R*-factors based on *F*^2^ are statistically about twice as large as those based on *F*, and *R*- factors based on ALL data will be even larger.

### Fractional atomic coordinates and isotropic or equivalent isotropic displacement parameters (Å^2^)

**Table:**

	*x*	*y*	*z*	*U*_iso_*/*U*_eq_	
O1	0.46453 (5)	0.76202 (15)	0.40473 (7)	0.0576 (3)	
O2	0.64520 (4)	0.88611 (14)	0.58892 (7)	0.0528 (3)	
O3	0.69778 (5)	1.07722 (17)	0.52182 (8)	0.0604 (4)	
N1	0.38948 (5)	0.63069 (15)	0.42694 (7)	0.0411 (3)	
N2	0.37762 (5)	0.53986 (16)	0.49225 (8)	0.0418 (3)	
N3	0.55797 (5)	0.72973 (16)	0.59800 (8)	0.0461 (3)	
N4	0.55879 (5)	0.81534 (15)	0.52518 (8)	0.0400 (3)	
C1	0.35469 (6)	0.63351 (18)	0.34260 (9)	0.0371 (3)	
C2	0.32860 (6)	0.49921 (19)	0.30573 (10)	0.0431 (4)	
H2	0.3357	0.4037	0.3339	0.052*	
C3	0.29185 (6)	0.5090 (2)	0.22647 (11)	0.0490 (4)	
H3	0.2730	0.4203	0.2022	0.059*	
C4	0.28296 (6)	0.6486 (2)	0.18339 (10)	0.0504 (4)	
H4	0.2579	0.6546	0.1303	0.060*	
C5	0.31115 (7)	0.7800 (2)	0.21888 (11)	0.0497 (4)	
H5	0.3061	0.8736	0.1886	0.060*	
C6	0.34670 (6)	0.77364 (19)	0.29874 (10)	0.0440 (4)	
H6	0.3652	0.8629	0.3230	0.053*	
C7	0.44219 (6)	0.68619 (19)	0.45316 (9)	0.0405 (4)	
C8	0.46208 (6)	0.63199 (18)	0.53974 (9)	0.0379 (3)	
C9	0.42086 (6)	0.54514 (18)	0.56011 (9)	0.0391 (4)	
C10	0.51460 (6)	0.6533 (2)	0.59932 (9)	0.0455 (4)	
H10	0.5176	0.5990	0.6505	0.055*	
C11	0.60540 (6)	0.89180 (17)	0.52552 (9)	0.0381 (3)	
C12	0.60884 (6)	0.98077 (18)	0.44780 (9)	0.0384 (4)	
C13	0.65628 (6)	1.06824 (19)	0.45021 (10)	0.0423 (4)	
C14	0.66193 (7)	1.1489 (2)	0.37797 (11)	0.0520 (4)	
H14	0.6933	1.2075	0.3802	0.062*	
C15	0.62176 (8)	1.1429 (2)	0.30318 (11)	0.0545 (4)	
H15	0.6261	1.1964	0.2548	0.065*	
C16	0.57499 (8)	1.0581 (2)	0.29950 (11)	0.0567 (5)	
H16	0.5478	1.0541	0.2487	0.068*	
C17	0.56846 (7)	0.9792 (2)	0.37078 (10)	0.0495 (4)	
H17	0.5364	0.9235	0.3678	0.059*	
C18	0.32105 (7)	0.5084 (2)	0.49384 (11)	0.0549 (5)	
H18A	0.3183	0.5003	0.5524	0.082*	
H18B	0.3094	0.4122	0.4644	0.082*	
H18C	0.2982	0.5921	0.4658	0.082*	
C19	0.42026 (7)	0.4633 (2)	0.64200 (10)	0.0536 (4)	
H19A	0.3935	0.3815	0.6307	0.080*	
H19B	0.4113	0.5365	0.6818	0.080*	
H19C	0.4556	0.4193	0.6662	0.080*	
H4N	0.5272 (7)	0.812 (2)	0.4759 (11)	0.064*	
H3O	0.6855 (9)	1.012 (2)	0.5610 (13)	0.080*	

### Atomic displacement parameters (Å^2^)

**Table:**

	*U*^11^	*U*^22^	*U*^33^	*U*^12^	*U*^13^	*U*^23^
O1	0.0432 (6)	0.0856 (9)	0.0391 (6)	−0.0239 (6)	0.0004 (5)	0.0156 (6)
O2	0.0401 (6)	0.0670 (8)	0.0431 (6)	−0.0113 (6)	−0.0058 (5)	0.0095 (5)
O3	0.0418 (7)	0.0787 (9)	0.0547 (8)	−0.0157 (6)	0.0001 (6)	0.0107 (6)
N1	0.0359 (7)	0.0532 (8)	0.0339 (7)	−0.0077 (6)	0.0080 (5)	0.0031 (6)
N2	0.0380 (7)	0.0511 (8)	0.0388 (7)	−0.0065 (6)	0.0141 (6)	0.0016 (6)
N3	0.0427 (8)	0.0572 (9)	0.0342 (7)	−0.0076 (7)	0.0010 (6)	0.0060 (6)
N4	0.0358 (7)	0.0470 (8)	0.0331 (7)	−0.0045 (6)	0.0001 (5)	0.0042 (6)
C1	0.0298 (7)	0.0484 (9)	0.0334 (7)	−0.0019 (6)	0.0083 (6)	−0.0051 (7)
C2	0.0414 (9)	0.0445 (9)	0.0440 (9)	−0.0030 (7)	0.0117 (7)	−0.0051 (7)
C3	0.0409 (9)	0.0573 (11)	0.0488 (10)	−0.0091 (8)	0.0111 (8)	−0.0149 (8)
C4	0.0360 (9)	0.0707 (13)	0.0406 (9)	0.0002 (8)	0.0021 (7)	−0.0055 (8)
C5	0.0464 (10)	0.0539 (11)	0.0466 (9)	0.0041 (8)	0.0073 (8)	0.0046 (8)
C6	0.0422 (9)	0.0451 (10)	0.0439 (9)	−0.0052 (7)	0.0088 (7)	−0.0046 (7)
C7	0.0368 (8)	0.0485 (9)	0.0346 (8)	−0.0068 (7)	0.0058 (6)	−0.0012 (7)
C8	0.0376 (8)	0.0437 (9)	0.0325 (8)	−0.0018 (7)	0.0084 (6)	−0.0025 (6)
C9	0.0407 (8)	0.0431 (9)	0.0354 (8)	−0.0003 (7)	0.0130 (7)	−0.0025 (6)
C10	0.0451 (9)	0.0570 (11)	0.0322 (8)	−0.0048 (8)	0.0053 (7)	0.0058 (7)
C11	0.0341 (8)	0.0388 (8)	0.0377 (8)	−0.0008 (6)	0.0014 (6)	−0.0019 (6)
C12	0.0361 (8)	0.0382 (9)	0.0387 (8)	0.0016 (6)	0.0046 (7)	0.0006 (6)
C13	0.0382 (8)	0.0425 (9)	0.0439 (9)	−0.0007 (7)	0.0058 (7)	−0.0001 (7)
C14	0.0512 (10)	0.0506 (11)	0.0555 (10)	−0.0083 (8)	0.0157 (8)	0.0043 (8)
C15	0.0652 (12)	0.0527 (11)	0.0464 (10)	−0.0001 (9)	0.0155 (9)	0.0086 (8)
C16	0.0574 (11)	0.0642 (12)	0.0414 (10)	−0.0053 (9)	−0.0017 (8)	0.0081 (8)
C17	0.0448 (9)	0.0551 (11)	0.0436 (9)	−0.0078 (8)	0.0011 (7)	0.0065 (8)
C18	0.0400 (9)	0.0760 (13)	0.0515 (10)	−0.0125 (9)	0.0167 (8)	0.0009 (9)
C19	0.0559 (11)	0.0632 (12)	0.0440 (9)	−0.0041 (9)	0.0165 (8)	0.0081 (8)

### Geometric parameters (Å, °)

**Table:**

O1—C7	1.2469 (18)	C6—H6	0.9300
O2—C11	1.2445 (17)	C7—C8	1.434 (2)
O3—C13	1.3553 (18)	C8—C9	1.381 (2)
O3—H3O	0.95 (2)	C8—C10	1.445 (2)
N1—C7	1.3785 (19)	C9—C19	1.492 (2)
N1—N2	1.3940 (17)	C10—H10	0.9300
N1—C1	1.4225 (18)	C11—C12	1.481 (2)
N2—C9	1.3417 (19)	C12—C17	1.397 (2)
N2—C18	1.4588 (19)	C12—C13	1.405 (2)
N3—C10	1.280 (2)	C13—C14	1.385 (2)
N3—N4	1.3830 (17)	C14—C15	1.370 (2)
N4—C11	1.3450 (19)	C14—H14	0.9300
N4—H4N	0.978 (18)	C15—C16	1.375 (2)
C1—C6	1.381 (2)	C15—H15	0.9300
C1—C2	1.384 (2)	C16—C17	1.371 (2)
C2—C3	1.382 (2)	C16—H16	0.9300
C2—H2	0.9300	C17—H17	0.9300
C3—C4	1.372 (2)	C18—H18A	0.9600
C3—H3	0.9300	C18—H18B	0.9600
C4—C5	1.378 (2)	C18—H18C	0.9600
C4—H4	0.9300	C19—H19A	0.9600
C5—C6	1.375 (2)	C19—H19B	0.9600
C5—H5	0.9300	C19—H19C	0.9600
			
C13—O3—H3O	102.6 (13)	C8—C9—C19	129.08 (14)
C7—N1—N2	109.30 (12)	N3—C10—C8	134.81 (14)
C7—N1—C1	127.67 (12)	N3—C10—H10	112.6
N2—N1—C1	121.87 (12)	C8—C10—H10	112.6
C9—N2—N1	108.03 (12)	O2—C11—N4	121.06 (14)
C9—N2—C18	126.78 (13)	O2—C11—C12	120.45 (13)
N1—N2—C18	120.37 (13)	N4—C11—C12	118.47 (13)
C10—N3—N4	118.23 (13)	C17—C12—C13	117.59 (14)
C11—N4—N3	116.54 (12)	C17—C12—C11	124.04 (14)
C11—N4—H4N	124.0 (11)	C13—C12—C11	118.34 (13)
N3—N4—H4N	119.4 (11)	O3—C13—C14	117.70 (14)
C6—C1—C2	120.59 (14)	O3—C13—C12	122.12 (14)
C6—C1—N1	118.62 (13)	C14—C13—C12	120.18 (15)
C2—C1—N1	120.77 (14)	C15—C14—C13	120.58 (16)
C3—C2—C1	119.07 (15)	C15—C14—H14	119.7
C3—C2—H2	120.5	C13—C14—H14	119.7
C1—C2—H2	120.5	C14—C15—C16	120.14 (16)
C4—C3—C2	120.46 (15)	C14—C15—H15	119.9
C4—C3—H3	119.8	C16—C15—H15	119.9
C2—C3—H3	119.8	C17—C16—C15	119.99 (16)
C3—C4—C5	119.91 (15)	C17—C16—H16	120.0
C3—C4—H4	120.0	C15—C16—H16	120.0
C5—C4—H4	120.0	C16—C17—C12	121.51 (16)
C6—C5—C4	120.50 (16)	C16—C17—H17	119.2
C6—C5—H5	119.8	C12—C17—H17	119.2
C4—C5—H5	119.8	N2—C18—H18A	109.5
C5—C6—C1	119.35 (15)	N2—C18—H18B	109.5
C5—C6—H6	120.3	H18A—C18—H18B	109.5
C1—C6—H6	120.3	N2—C18—H18C	109.5
O1—C7—N1	122.57 (13)	H18A—C18—H18C	109.5
O1—C7—C8	131.79 (14)	H18B—C18—H18C	109.5
N1—C7—C8	105.59 (13)	C9—C19—H19A	109.5
C9—C8—C7	107.25 (13)	C9—C19—H19B	109.5
C9—C8—C10	122.31 (14)	H19A—C19—H19B	109.5
C7—C8—C10	130.42 (14)	C9—C19—H19C	109.5
N2—C9—C8	109.73 (13)	H19A—C19—H19C	109.5
N2—C9—C19	121.19 (14)	H19B—C19—H19C	109.5
			
C7—N1—N2—C9	3.35 (17)	N1—N2—C9—C19	178.01 (14)
C1—N1—N2—C9	171.97 (13)	C18—N2—C9—C19	22.9 (2)
C7—N1—N2—C18	160.36 (14)	C7—C8—C9—N2	1.00 (18)
C1—N1—N2—C18	−31.0 (2)	C10—C8—C9—N2	−177.66 (14)
C10—N3—N4—C11	−178.83 (15)	C7—C8—C9—C19	−179.73 (16)
C7—N1—C1—C6	−52.2 (2)	C10—C8—C9—C19	1.6 (3)
N2—N1—C1—C6	141.46 (14)	N4—N3—C10—C8	−0.2 (3)
C7—N1—C1—C2	128.96 (17)	C9—C8—C10—N3	−177.33 (18)
N2—N1—C1—C2	−37.4 (2)	C7—C8—C10—N3	4.4 (3)
C6—C1—C2—C3	−3.9 (2)	N3—N4—C11—O2	−0.7 (2)
N1—C1—C2—C3	175.00 (13)	N3—N4—C11—C12	178.00 (13)
C1—C2—C3—C4	2.6 (2)	O2—C11—C12—C17	172.71 (15)
C2—C3—C4—C5	0.5 (2)	N4—C11—C12—C17	−6.0 (2)
C3—C4—C5—C6	−2.4 (2)	O2—C11—C12—C13	−5.1 (2)
C4—C5—C6—C1	1.1 (2)	N4—C11—C12—C13	176.17 (14)
C2—C1—C6—C5	2.0 (2)	C17—C12—C13—O3	179.98 (15)
N1—C1—C6—C5	−176.85 (14)	C11—C12—C13—O3	−2.1 (2)
N2—N1—C7—O1	175.00 (15)	C17—C12—C13—C14	−0.1 (2)
C1—N1—C7—O1	7.2 (3)	C11—C12—C13—C14	177.82 (15)
N2—N1—C7—C8	−2.66 (17)	O3—C13—C14—C15	179.16 (16)
C1—N1—C7—C8	−170.43 (14)	C12—C13—C14—C15	−0.8 (3)
O1—C7—C8—C9	−176.30 (18)	C13—C14—C15—C16	0.8 (3)
N1—C7—C8—C9	1.05 (17)	C14—C15—C16—C17	0.1 (3)
O1—C7—C8—C10	2.2 (3)	C15—C16—C17—C12	−1.0 (3)
N1—C7—C8—C10	179.56 (16)	C13—C12—C17—C16	1.0 (3)
N1—N2—C9—C8	−2.65 (17)	C11—C12—C17—C16	−176.83 (16)
C18—N2—C9—C8	−157.76 (16)		

### Hydrogen-bond geometry (Å, °)

**Table:**

*D*—H···*A*	*D*—H	H···*A*	*D*···*A*	*D*—H···*A*
O3—H3O···O2	0.95 (2)	1.62 (2)	2.5076 (17)	155 (2)
N4—H4N···O1	0.978 (18)	1.763 (18)	2.7140 (16)	163.3 (16)
